# Reducing Allostatic Load in Depression and Anxiety Disorders: Physical Activity and Yoga Practice as Add-On Therapies

**DOI:** 10.3389/fpsyt.2020.00501

**Published:** 2020-06-04

**Authors:** Luciana D'Alessio, Guido Pablo Korman, Mercedes Sarudiansky, Laura Ruth Guelman, Laura Scévola, Alejandra Pastore, Amilcar Obregón, Emilio J. A. Roldán

**Affiliations:** ^1^Universidad de Buenos Aires, Facultad de Medicina, IBCN-CONICET, Buenos Aires, Argentina; ^2^Universidad de Buenos Aires, Hospital Ramos Mejía, Buenos Aires, Argentina; ^3^Universidad de Buenos Aires, Facultad de Psicología, CAEA-CONICET, Buenos Aires, Argentina; ^4^Universidad de Buenos Aires, Facultad de Medicina, CEFYBO-CONICET, Buenos Aires, Argentina; ^5^Dirección Médica y Científica, Gador SA, Buenos Aires, Argentina

**Keywords:** chronic stress, neuroplasticity, neurogenesis, hippocampus, cortisol, noradrenaline, antidepressants, benzodiazepines

## Abstract

The allostatic load (AL) index constitutes a useful tool to objectively assess the biological aspects of chronic stress in clinical practice. AL index has been positively correlated with cumulative chronic stress (physical and psychosocial stressors) and with a high risk to develop pathological conditions (e.g., metabolic syndrome, cardiovascular pathology, inflammatory disorders) and the so-called stress-related psychiatric disorders, including anxiety and depressive disorders. Chronic stress has negative effects on brain neuroplasticity, especially on hippocampal neurogenesis and these effects may be reversed by antidepressant treatments. Several evidences indicate that non-pharmacological interventions based on physical activity and yoga practice may add synergizing benefits to classical treatments (antidepressant and benzodiazepines) for depression and anxiety, reducing the negative effects of chronic stress. The aim of this review is to provide a general overview of current knowledge on AL and chronic stress in relation to depression and anxiety, physical activity and yoga practice.

## Introduction

The concept of allostatic load (AL) index, has been introduced by Bruce McEwen and Stellar in 1993 to objectively assess the biological consequences of chronic stress in the body and the brain (1). Psychosocial factors related to poverty and health risk behaviors across the lifespan have been associated with higher AL index (2, 3). AL index has been positively correlated with cumulative chronic stress (physical and psychosocial stressors) and with a high risk to acquire medical conditions (e.g., metabolic syndrome, cardiovascular pathology, inflammatory disorders) (4–8). Moreover, chronic stress and AL overload may contribute to the expression of the so-called stress-related psychiatric disorders, especially represented by anxiety and depressive disorders (5, 9–12). Furthermore, chronic stress through hypercortisolemia can induce negative effects on brain neuroplasticity, especially on hippocampal neurogenesis (13–16) and these effects may be reversed by antidepressant treatments (17, 18).

Evidences based on experimental data and studies in human patients indicate that physical activity and yoga practice might bring about benefits not only in adult hippocampal neurogenesis and neuroplasticity (19), but also on stress related psychiatric symptoms (anxiety and depression) (20, 21) as well as on AL index (22, 23). Non-pharmacological interventions and classical treatments (psychotherapeutic and pharmacological approaches) may have synergistic effects on brain function and clinical symptoms which helps building the idea of managing depression and anxiety disorders using combined treatments (19, 21). The implementation of these strategies as add-on therapy may optimize the pharmacological treatment reducing the risk of adverse side effects. Therefore, the aims of this review are to provide an overview of the biology aspects of AL and chronic stress in reference to depression and anxiety, and to describe the current knowledge on the non-pharmacological interventions based on physical activity and yoga practice upon AL, depression and anxiety.

## The Allostasis Concept and the Allostatic Load Index

The term allostasis refers the ability to achieve stability through changes in response to stress and/or stressful events (4). The most important systems involved in the stress response are the hypothalamic-pituitary-adrenal (HPA) axis, the autonomic nervous system and the inflammatory system, with their respective biochemical mediators, the glucocorticoids, the catecholamines (adrenaline and noradrenaline) and the cytokines. These compounds produce physiologic effects that are adaptive in the short term, however they can be harmful in the long term (chronic stress) (4, 5, 10, 13).

The AL index was proposed with the aim of measuring the cumulative influence of stress on health through a series of clinical and biochemical indicators of the main systems potentially affected (4, 6). Essentially, the AL index constitutes a marker of cumulative biological risk of chronic stress which describes the additive effects of multiple clinical situations that may accelerate the vulnerability to diseases reducing resilience (9, 10, 24–26). The AL index was found to be a good predictor of mortality and physical functioning (27). The *clinimetric* definition of AL overload, firstly introduced by Feinstein in 1987 to define clinical measurements related to chronic stress, was recently described by Fava and McEwen et al. in 2019 (27). These authors proposed two main criteria: the presence of a current source of distress added to previous symptoms or functioning impairment (27).

AL index includes different biological markers divided in two main groups; 1-Primary mediators: represented by stress hormones (adrenaline, noradrenaline, and cortisol) in combination with inflammatory cytokines (e.g., interleukin-6, tumor necrosis factor), 2- Secondary mediators: represented by biomarkers of metabolic, cardiovascular, and immune systems that compensate primary mediators alterations [e.g., insulin, glucose, total cholesterol, high density lipoprotein cholesterol, triglycerides, visceral fat depositing, systolic and diastolic blood pressure, fibrinogen, c-reactive protein; (2, 8, 28)]. Different authors have applied the AL index in population-based studies and/or in clinical trials and studied the association with depression and anxiety disorders (8, 11, 28–32). **Table 1** summarizes the AL index and the cut off points analyzed in a previous study performed by our group, in patients with general anxiety disorder (12, 32).

**Table 1:** Allostatic load parameters and cut-off points.

Affected systems	Allostatic Load Parameters	Cut-off points (for AL index) (32)
**Primary mediators**		
HPA-Cortisol	Cortisol	>32nM Salivary
	DHEA (dehydro-epi-androsterone)	<80 ng/ml in men <35 ng/ml in women (plasma)
Autonomic System	Noradrenaline	>100pg/ml (plasma)
	MHPG (methoxy-hydroxy-phenylglycol)	>2,750nM (salivary)
**Secondary mediators**		
Cardiovascular	Systolic blood pressure	>140 mmHg
	Diastolic blood pressure	>90 mmHg
Metabolism: Lipids and adipose tissue deposition	Body Mass Index	>25
	Waist-hip ratio	>1 in men >0.8 in women
	Total cholesterol	>200 mg/dl (plasma)
	LDL (low density lipoprotein)	>120 mg/dl (plasma)
	HDL (high density lipoprotein)	<37 mg/dl (plasma)
	Total cholesterol/HDL ratio	>3.5 mg/dl (plasma)
Metabolism: Glucids, proteins and renal function	Glycated hemoglobin	>6mg/dl (plasma)
	Creatinine	>1.2mg/dl (plasma)
	Albumin	<3.5 g/dl (plasma)
Immune System	C-reactive protein	>7.1mg % (plasma)
	Fibrinogen	>400mg/dl (plasma)

## Depression, Anxiety, and AL

The relationship between chronic stress, depression and anxiety has been extensively described (5, 9, 10, 13). Experimental models showed that stress hormones (glucocorticoids) acting on the brain, may modify emotional arousal, predisposing to depression- and anxiety-like behavior (33). Furthermore, chronic stress models in rodents can cause changes in brain neuroplasticity and changes in behavior consistent with depressive- and anxiety-like symptoms (5, 9, 10, 13, 33–36).

In humans, the administration of exogenous glucocorticoids may produce psychiatric adverse effects, such as steroid psychoses and depression. These symptoms may be blocked by the glucocorticoid antagonist mifepristone (37, 38). In patients with major depressive disorder, cortisol levels are elevated, the diurnal rhythm is distorted and the HPA axis is resistant to suppression by dexamethasone (9, 33, 37, 38). Moreover, higher AL index has been associated with higher levels of depressive symptoms in many studies (8, 11) (28–30, 39). Compared with patients with personality disorders, emergency psychiatric patients primarily diagnosed with bipolar, depressive, or anxiety disorders showed altered AL parameters including cortisol, interleukin-6, systolic blood pressure, and heart rate (11). Also, higher AL index were significantly associated with depressive symptoms (29) showing variations according to sex, gender, race, and sociocultural factors (30, 31).

Cardiovascular parameters of AL index were frequently altered in patients with depression (11, 28, 29). Longitudinal cohort studies showed increase risk of cardiovascular morbidity and mortality among patients with comorbid depression (40). In a recent study, Gillespie et al. analyzed the role of AL and depression in relation to coronary heart disease. Greater depressive symptomatology was associated with higher metabolic, cardiovascular, and immune AL parameters, however coronary heart disease was associated to depression, only in male population (28). Obesity, metabolic abnormalities and higher expression of inflammatory markers were also frequently observed in patients with depression (23, 30, 41). Compared with age- and gender-matched control groups, individuals with major depression had higher metabolic syndrome prevalence, hyperglycaemia, and hypertriglyceridemia (41).

Regarding anxiety, high levels of cortisol and CRH (corticotrophin releasing hormone), have been associated to fear states and anxiety-like behavior in experimental models (13, 33). Disturbances in the HPA with hypercortisolemia were reported among patients with anxiety disorders, in patients with panic disorder (42) and in patients with general anxiety disorder (32, 43). In addition, sympathetic hyperactivity with high levels of methoxyhidroxiphenilglicol (MHPG) (the main metabolite of noradrenaline) was also reported in patients with anxiety disorder (32, 44, 45).

Sex and age related differences in psychiatric symptoms and AL parameters were also described in the literature (28–30, 32, 46). Firstly, a higher prevalence and severity of depression and anxiety disorders has been reported in women compared to men (29, 32, 47). Biological differences have been proposed to explain such differences since experimental studies in rodents suggest sex differences in the neural remodeling pattern after stress (10, 48). However, psychosocial influences cannot be excluded as women are also more exposed to psychosocial stress and trauma (30, 49, 50). Women in fertile age are less prone to cardiovascular disease and mortality than men (28, 31, 51, 52). In a previous study of our group in patients with anxiety disorders, women showed higher anxiety levels than men, with a better profile in many individual AL variables, particularly cardiovascular (systolic blood pressure), obesity (body mass index), and lipids with higher HDL (high density lipoprotein cholesterol) levels (32). On the opposite, AL inflammatory parameters tended to be higher in women (30, 46, 52). Regarding age factors, elderly patients were more vulnerable to stress-related disorders and expressed higher levels of cumulative AL (2, 6, 7, 32).

## Chronic Stress and Hippocampal Neuroplasticity: The Role of Antidepressant Treatments

The hippocampus has been in the focus of stress research especially after the finding that glucocorticoids receptors are abundantly expressed in hippocampal cells (53). A large body of evidence in rodents demonstrated that chronic stress, *via* elevated levels of glucocorticoids, can affect both hippocampal structure and function (16, 33, 54) and can also induce depressive-like behavior (18). Adult hippocampal neurogenesis (the brain capacity to develop new neurons during adult life) (55), decrease with age and is especially affected by chronic stress (15, 56, 57).

Hippocampal structure and function might be altered in patients with depression. Reductions in hippocampal volume based on magnetic resonance imaging studies have been consistently documented in patients with major depression (58, 59). For example, patients suffering from recurrent depressive episodes showed lower hippocampal volume (60, 61). Similarly, in Cushing's disease, the duration of the illness predicts a progressive reduction in the hippocampus volume, which was determined by structural magnetic resonance imaging (62). Besides, the atrophy of the hippocampus has also been reported among patients with anxiety-related disorders and post-traumatic stress disorder (63). Moreover, depression is a common comorbidity in patients with resistant temporal lobe epilepsy and hippocampal sclerosis (14, 64, 65).

In humans, adult hippocampal neurogenesis occurs in the hippocampal dentate gyrus, and newborn neurons can be produced from neural stem cells which can be classified based on their distinctive morphology as radial glia-like (66) and non-radial glial cells (67). Chronic physical stressors, chronic psychosocial stressors, and chronic unpredictable stressors, can inhibit one or more phases of the adult hippocampal neurogenesis process (15). Neurotransmitters systems that regulate adult hippocampal neurogenesis might be affected by chronic stress: The enhance of glutamate release *via* NMDA (N-methyl-D-aspartate receptor), the reduction of GABA (Gamma aminobutyric acid) levels, the reduction of 5HT (serotonin) levels and the down regulation of 5HT1A (serotonin receptor 1A; stimulation of 5HT1A is pro-neurogenic), and the alterations in noradrenaline, dopamine, and endocannabinoids were described (18, 57, 68). Stress can also reduce the expression of neurotrophic factors involved in adult hippocampal neurogenesis regulation (e.g., brain-derived neurotrophic factor, insulin-like growth factor-1, neuregulin-1; 69, 70). Neurotrophic factors have been shown to increase adult hippocampal neurogenesis and also modulate antidepressant-related effects and behavior in experimental models (18, 71).

Adult hippocampal neurogenesis is involved in mediating the response to antidepressant drugs (18, 72). The suppression of adult hippocampal neurogenesis achieved by chronic stress can be prevented by administering antidepressants (72, 73) which reverse the inhibitory effect of stress after 3–4 weeks of treatment (time course of maturation for newly generated neurons) (17, 72–75). Antidepressants require of adult hippocampal neurogenesis mechanisms to be effective (57, 76) and involve the activation of glucocorticoid receptors in the hippocampus (77). The stimulation of adult hippocampal neurogenesis has been considered a promising property for identifying new antidepressant targets. At the moment almost all antidepressant treatments including pharmacotherapy and behavioral interventions, proved to stimulate adult hippocampal neurogenesis (15, 57, 78).

## Chronic Stress and Anxiety: Role of Anxiolytics Treatments

During stress response sympathetic system is simultaneously activated with HPA system, and both noradrenaline and cortisol are released, amplifying the emotional response (4, 5, 10, 13). Anxiety has been proposed as an undifferentiated form of fear or rage, discharged by noradrenaline and cortisol (79). Anxiolytic drugs acting through a positive allosteric modulation of GABA-A receptor (benzodiazepines), reduce the sympathetic discharge leading to lower plasma levels of catecholamines (80–82) and lower salivary levels of MHPG (44, 45, 83, 84). Benzodiazepines, particularly those of high-potency such as alprazolam, have demonstrated anti-hypertensive pleiotropic properties in patients with high blood pressure without affecting the heart rate (81, 85). In our previous work (32), low doses of alprazolam during 12 weeks reduced anxiety levels and the total AL index. We observed a significant reduction on salivary levels of MHPG and on systolic blood pressure (12, 32). As well pharmacotherapy with benzodiazepines in association to psychotherapy demonstrated to be effective in reducing anxiety and AL parameters (86), the risk of inducing pharmacological dependence and/or cognitive adverse events should be considered requiring continuous monitoring (87–89).

## Non-Pharmacological Strategies, Physical Activity, and Yoga Practice

### Physical Activity

Many studies based on experimental research in rodents demonstrated that physical activity interventions may promote hippocampal neuroplasticity, counteracting the negative effects of chronic stress on the brain (15, 19, 90–95). Models of aerobic exercise potently stimulate adult hippocampal neurogenesis, inducing higher rates of proliferation and/or survival of newborn cells (15, 90). Also, higher synaptic plasticity and higher levels of BDNF (Brain Derived Neurotrophic Factor), were observed in hippocampus, after voluntary and involuntary exercise (91, 92). Additionally, the use of combined treatments (e.g., aerobic exercise and antidepressant treatments), has stronger effect on depressive behavior and on BDNF up-regulating, than antidepressant treatment or aerobic exercise acting individually (93–95).

In humans, neuroimaging studies using MRI (Magnetic Resonance Image) demonstrated that physical activity increased the hippocampal volume and positively correlated with better performances on hippocampus dependent tasks (episodic memory) (96). Physical activity can enhance some indirect signs of neuroplasticity (e.g., increased cerebral blood flow or BDNF plasma concentration) (96). Plasma levels of BDNF have been proposed as a potential biomarker of depression treatment. Serum BDNF is decreased in depressed patients and can be normalized with antidepressant treatment together with moderate to intense physical activity (97, 98).

Physical activity has been considered a non-stigmatizing intervention with few side effects and has been associated with the relief of depression and anxiety symptoms in different studies (20, 21, 99, 100). Different types of physical activity (aerobic, stretching, leisure-physical activities, dance, yoga *asanas*), have been associated with lower depression and anxiety prevalence in population-based studies (23, 100–102), with a better outcome in patients with depressive disorders (103–106) and with lower scores of self-reported stress, anxiety, and depression scales in healthy subjects (107–109). Physical activity may prevent the development of depression during the follow up according prospective studies (23, 100, 102), can reduce de the risk of anxiety (110) and may enhance the cognitive performance (97) contributing with antidepressant drugs and/or cognitive-behavioral therapy (111). In a meta-analysis of relevant randomized controlled clinical trials, aerobic exercise showed moderate to large effects for patients with major depression and favoured over classical psychological treatments or antidepressants drugs (20). Physical activity may act as an adjunctive treatment to traditional medication and psychotherapy, resulting in a more effective approach toward relief of psychiatric symptoms and AL (19, 20, 99, 105).

**Table 2** resumes the main recent studies that have analyzed physiological AL parameters and the effects of physical activity (and/or yoga *asanas*) on depression and anxiety outcome. All resumed studies, population-based studies and clinical trials, found a positive effect on depression and anxiety symptoms outcome in healthy subjects and in patients with depression. Although the total AL index was not measured, some of these studies analyzed certain AL parameters outcome. Obesity factors were positively associated with depression and negatively with physical activity (23). A lower fitness level in cardiopulmonary exercises was observed in patients with depression (104). Minor resting pulse was associated with physical activity and lower risk to develop depression in healthy subjects (100). Also, lower heart rate and diastolic blood pressure and higher HDL cholesterol, were observed among inpatients with major depressive disorder who underwent to a physical activity-training program (105). Regarding the effects of physical activity on AL parameters sex differences were also reported (106, 112). Women showed higher levels of c-reactive protein, which positively correlated with measures of adiposity and inversely associated with fitness, suggesting that adiposity may play a more substantial role in inflammation in women (106). Although these preliminary findings are promising, larger and adequately powered randomised controlled trials are needed to better evaluate the combined effect of physical activity and classical treatments on allostatic load and on depression and anxiety.

**Table 2:** Description of the studies that have been analyzed the physiological AL parameters and the effects of physical activity and yoga asanas on anxiety and depression outcome.

Study	Type of study	Study participants	Physical activity	Concomitant treatment	AL parameters measured	Depression/Anxiety outcome	Main conclusion
**Physical Activity (Aerobic Exercises, strengthening activities, leisure - physical activity surveys)**							
Bennie et al. (101) USA	Cross-sectional data analyses from health surveillance surveys (2011–2017).	n=1,477,981 adults (≥18 years). n=286,325 (18.0%) had depression.	Self reported physical activity survey. Physical activity was classified as aerobic and non-aerobic, and moderate intensity or vigorous intensity.	Not reported	Body Mass Index Diabetes Hypertension Arthritis Subjects were analyzed in stratified sub- groups to reduce cofounders. AL outcomes were not reported.	All physical activity combinations were associated with lower prevalence of depression	Lowest prevalence of depression was shown for those combining aerobic physical activity, or muscle strengthening activities, ≥2 days/week.
Fernandez-Montero et al. (102) Spain	Prospective study of physical activity and risk of depression	n=15,488 adults, follow-up of 10.5 years.	Leisure physical activity questionnaire (hours/week).	Not reported	Hypertension, Diabetes mellitus Weight (Analyzed as cofounders).	A total of 870 incident cases of depression	Participants with higher total leisure physical activity exhibited a lower risk of depression.
Gomes et al. (23) Brazil	Prospective study of adiposity, inflammatory markers, depression and anxiety	n=2,977 Cohort followed-up from birth until 18 and 22 years old	International Physical Activity Questionnaire (IPAQ)	No	Body mass index Fat mass index Waist circumference C-reactive protein IL-6	A bidirectional association between obesity and depression was found. The effect of obesity on depression was more consistent than on anxiety.	Depression, but not generalized anxiety disorder, was associated with adiposity. Decreased levels of physical activity may mediate the association between obesity and depression.
Harvey et al. (100) Norway	Prospective study about exercise and the prevention of depression.	n=33,908 Cohort of healthy adults were followed for 11 years.	Validated questioners of exercise.	No	Body Mass Index, resting pulse. Less exercise at baseline was associated with higher resting pulse.	22,564 individuals followed up, 1,578 (7.0%) developed depression and 1,972 (8.7%) anxiety.	Regular leisure-time exercise of any intensity provided protection against future depression but not anxiety.
Herbsleb et al. (104) Germany	Cardio-respiratory fitness and autonomic function in patients with major depressive disorder	n= 34 patients with depression and normal controls	International Physical Activity Questionnaire (IPAQ)	Conventional treatments (Antidepressants)	Hear rate, blood pressure, body fat, body mass index. Cardiopulmonary exercise test.	A negative correlation between depression and the IPAQ was found in patients with depression.	A lower fitness level in cardiopulmonary exercise test was observed in patients with depression.
Kerling et al. (105) Germany	Adjunctive exercise in depression: a randomized pilot trial	n=42 consecutive inpatients with major depressive disorder	Training program; three sessions per week, 45 minutes, moderate intensity compared to a group with usual treatment	Psychotherapy (100%) and antidepressants (75-77%)	Espiroergometry Lipids VO_2_ uptake Lactate levels	Exercise group showed lower heart rate, diastolic blood pressure, waist circumference, and higher HDL cholesterol and depression improvement.	Exercise improves the outcome of inpatient treatment of moderate to severe depression.
Philippot et al. (109) Belgium	A pilot randomised trial of physical exercise on depression and anxiety symptoms	n=27 Healthy pre-adolescents age=9-11 years.	5-week period of intensive or low-to moderate exercises, four times a week.	No	Body mass index, body fat. No significant differences were found after treatment.	Psychological self-reports of depression and anxiety were reduced.	The program focused on associating movement with pleasure, encouraged positive and non-competitive interactions between participants
Schuchet al. (103) Brazil	Effects of aerobic physical exercise as an add-on strategy; A randomized controlled trial	n=26 Severe depressed inpatients.	Aerobic physical exercise 16.5 kcal/kg/week, three times a week	Conventional treatments (pharmacotherapy and/or electroconvulsive therapy)	Weight. No AL outcomes were measured.	A significant reduction in depression symptoms scores was observed in the exercise group.	Physical exercise could be a feasible and effective add-on strategy for treatment of severe depressed inpatients.
Valentine et al. (106) USA	Sex differences between obesity, C-reactive protein, physical activity, depression and fatigue	n=127 community-dwelling older adults	Physical Activity Scale for the Elderly (PASE)	No	Body Mass % Fat Index C-Reactive Protein	Inflammation was positively associated with fatigue and depression, and inversely associated with physical activity and % fat in women.	Strategies to prevent fatigue and depression may differ in older women and men, especially with regard to inflammation, physical activity and adiposity.
**Yoga (*Hatha Yoga/asanas*)**							
Gothe et al. (107) USA	A randomized control study Effects of Yoga on executive function and stress	n = 118 healthy adults	An 8-week yoga intervention or a stretching control group.	No	Salivary cortisol	Executive functions, self-reported stress and anxiety	Yoga participants showed improvement in executive functions and showed an attenuated cortisol response.
West et al. (108) USA	Effects of *hatha yoga* and African dance on perceived stress and affect.	69 healthy undergraduate students.	Different interventions in 3 groups of patients: African dance, hatha yoga, and biology lecture (control group).	No	Salivary cortisol levels were decreased in yoga group.	Perceived Stress, Affect (positive and negative affect schedule).	African dance and *hatha yoga* significantly decreased perceived stress and negative affect, compared to the biology class.

### Yoga Practice

In the last years, yoga has become popular in western cultures and constitutes an interesting tool for stress management. Although yoga originated in India, different yoga styles are practiced in western societies at present. Most of the different styles contain physical postures (termed *asana* in Sanskrit), as well as breath control exercises (*pranayama*) and meditation (*dyana*) (113). A meta-analysis of randomised controlled trials performed on healthy subjects and on patients with medical conditions (e.g cardiopathy, hypertension, metabolic syndrome, obesity, breast cancer), showed that yoga practice may reduce physiological measures of stress, including cortisol, autonomic measures (systolic blood pressure, heart rate), cytokines, and lipid levels (21). Also, yoga practice showed positive effects on depression and on anxiety symptoms in pregnant women (114), improved the mood states in psychiatric inpatients (115) and had beneficial effects on depression outcome in patients with major depressive disorder (116–118).

Regarding AL parameters, attenuated and decreased levels of salivary cortisol after yoga practice were reported in clinical trials on healthy patients(107, 108). These results correlated with lower perceived stress and negative affect (108) and with better cognitive functions, lower self-reported stress, and minor anxiety levels (107) (**Table 2**). Clinical evidences showed that yoga *asanas* can modulate the autonomic nervous system inducing higher parasympathetic activity, lower blood pressure, lower heart rate, higher relaxation and approach behaviors (21, 119). In a recent study using magnetic resonance spectroscopy yoga practice has been associated to higher GABA levels in patients with major depressive disorder (118).

A combination of body awareness-yoga *asanas* and mindfulness meditation has been developed in a complete program for stress reduction (21) and has been proposed to clinicians as a safe and effective technique to reduce stress and anxiety in diverse patient populations (21, 120, 121) reducing stress, anxiety and depression, increasing the quality of life and well-being (107, 122–124). Yoga practice effectively decreases depressive and anxious symptomatology, although the neurobiological mechanisms involved are not totally elucidated (21). Regarding brain effects, neuroimaging studies demonstrated that hippocampus is activated during yoga practice (113, 125, 126). Moreover, a significantly greater hippocampal volume has been found in experienced yoga practitioners (113, 126, 127) and higher volumetric measures in different brain areas were positively correlated with the years of yoga practice (113).

## Conclusions

Non-pharmacological interventions such as physical activity and yoga practice may exert synergizing effects to antidepressant and anxiolytic treatments. The classical treatment of patients with anxiety disorders and depression is psychotherapy, but pharmacotherapy is recommended when psychiatric symptoms are severe enough to induce a significant functional impairment (86). Antidepressants and/or benzodiazepines are indicated (National for Health and Clinical Excellence, UK guidelines) to treat these disorders (86). However, pharmacological treatments may be associated with adverse side effects. Physical activity and yoga practice have been shown to elicit improvements in anxiety and depression symptoms conducting to a better social, physical, and affective well-being optimizing the pharmacological treatment duration and the risk of pharmacological adverse side effects. However, larger and adequately powered randomized controlled trials are needed to evaluate the combined effect of physical activity on allostatic load and on depression and anxiety. Also, is important to emphasize that future studies are needed to reveal the biological mechanisms involved in the therapeutic actions of physical activity and yoga, in particular on depression and anxiety. Furthermore, stratified interventions according to sex and age should be considered in the future to track the therapies toward an individualized schedule in agreement with the current medical practice.

## Author Contributions

LD'A, GK, MS, LG, and LS have been involved in drafting and revising the manuscript for intellectual content. AO, AP, and ER have made substantial contributions to the conception and design. LD'A and ER have given the final approval of the version to be published.

## Conflict of Interest

LD'A received a grant for participating in this project from Gador SA. AO, AP, and ER are employees of Gador SA.

The remaining authors declare that the research was conducted in the absence of any commercial or financial relationships that could be construed as a potential conflict of interest.
